# Tc-MDP bone scintigraphy in a case with sporodical tumoral calcinosis

**DOI:** 10.4103/0972-3919.63598

**Published:** 2010

**Authors:** Tulay Kacar Guveli, Mehmet Mulazimoglu, Muge Oner Tamam, Cüneyt Tamam, Tarik Tatoglu, Tevfik Ozpacaci

**Affiliations:** Department of Nuclear Medicine, Okmeydani Training and Research Hospital, Istanbul, Turkey; 1 Department of Orthopedics and Traumatology, Kasımpasa Military Hospital, Istanbul, Turkey

**Keywords:** Sporadical tumoral calcinosis, Tc-MDP bone scintigraphy, juxta-articular regions

## Abstract

Tumoral calcinosis is an uncommon and benign condition characterized by the presence of large calcific soft tissue deposits occurring predominantly in a periarticular location. It generally occurs as a complication of renal dialysis or trauma, and is rarely seen in familial and sporadic cases. Bone scintigraphy is a sensitive method for diagnosing tumoral calcinosis. A 28-year-old female patient with a history of operation due to tumoral calcinosis located bilateral hips, referred to our department. She had a tender palpable mass in the right knee and a fistulized incisional scar overlying the bilateral hip joints. A sporadic case of tumoral calcinosis with relapses was presented.

## INTRODUCTION

Tumoral calcinosis, is rare a clinical entity first described by Duret in 1899.[1] The term 'tumoral calcinosis' was first used by Inclan *et al*. in 1943.[2] It is a periarticular tumor-like calcified mass often occurring in the juxta-articular regions of the extremities. A sporadic case of tumoral calcinosis with relapses was presented.

## CASE REPORT

**Figure 1:**
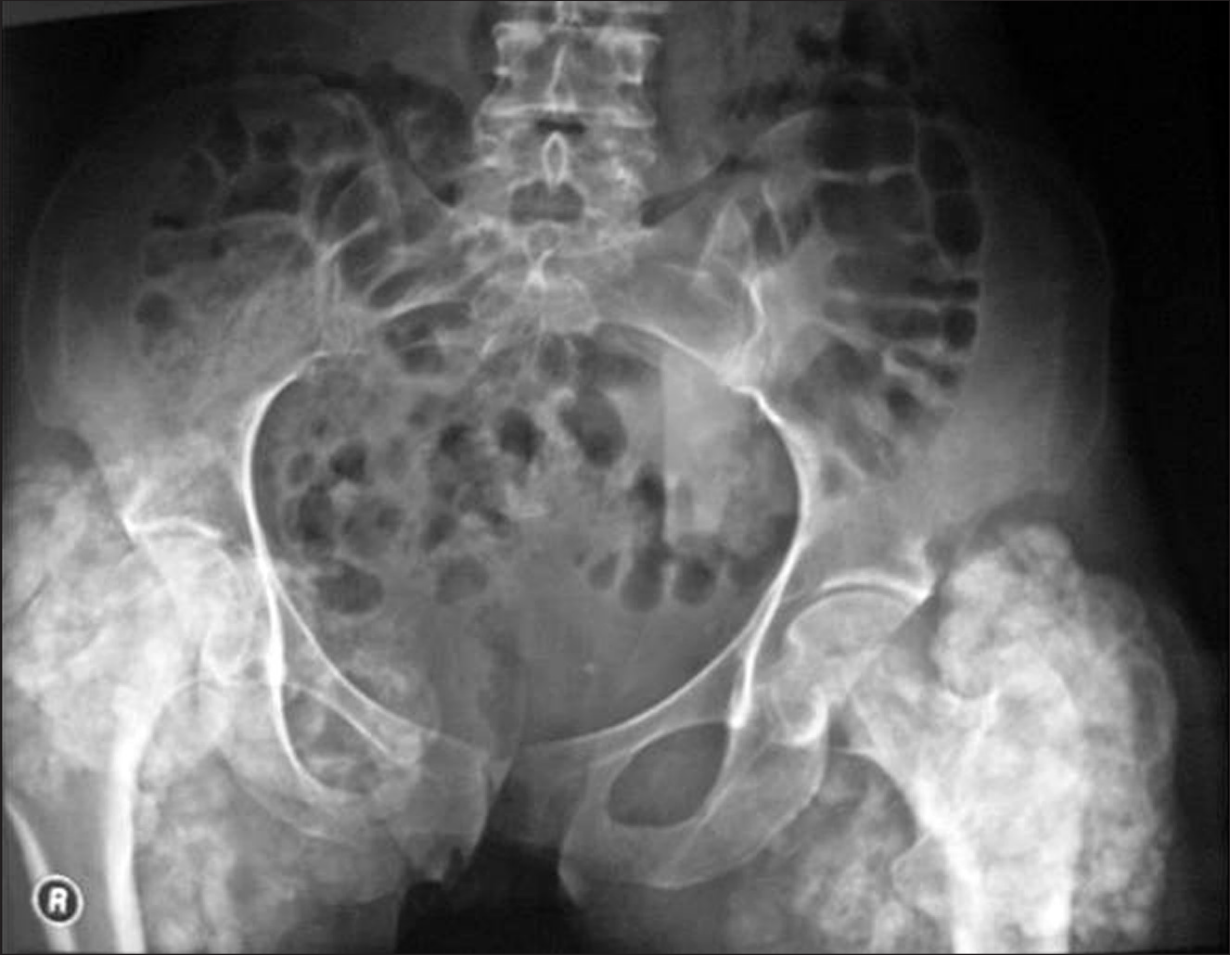
The anteroposterior pelvis graphy, determined multilobular dense nodular components in the periarticular soft tissue around the pelvis joint

**Figure 2:**
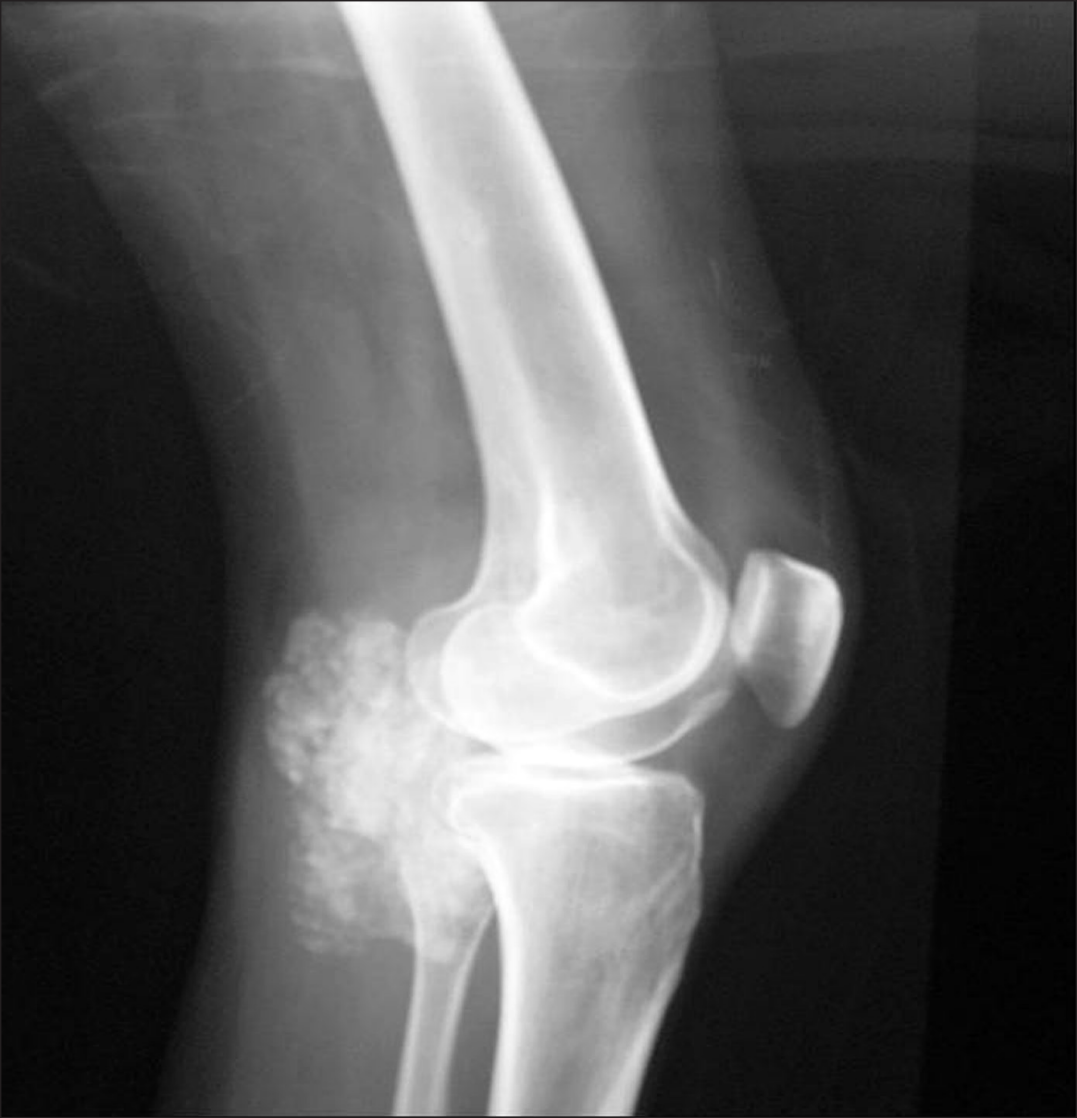
Radiographs of the right knee reveal calcified masses with lucent areas in the popliteal cavity of the knee joint

**Figure 3:**
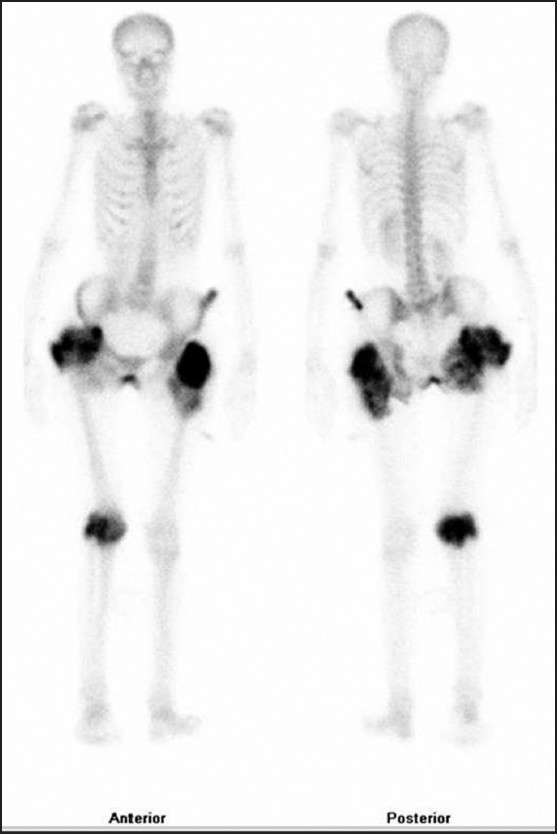
Tc-99m MDP whole body bone scintigraphy reveals increased uptake with heterogenic character in the hip, left iliac wing, and the right knee area

28-year-old female patient referred to our clinic with complaints of pain in both hips, a tender palpable mass in the flexor tendons of the right knee and drainage from the fistulized areas on the old incision line in the bilateral trochanteric area. In her medical history, she was first operated in 1993, with diagnosis of tumor calcinosis in the right hip. Later on she had two more operations, with the same diagnosis, on bilateral hips in 1997 and 2007. She had no history of trauma and no other systemic illness was elicited. Laboratory evaluation including serum calcium, phosphorus, and parathormone, and alkaline phosphatase levels were within normal limits. The anteroposterior pelvis graphy, determined multilobular dense nodular components in the periarticular soft tissue around the pelvis joint [Figure 1]. Radiographs of the right knee also revealed calcified masses with lucent areas in the popliteal cavity of the knee joint [Figure 2]. Tc-99m MDP whole body bone scintigraphy revealed an increased uptake with a heterogenic character, in the hip, left iliac wing, and the flexor aspect of the right knee area [Figure 3].

## DISCUSSION

Tumoral calcinosis is a rare disease characterized by large calcific soft tissue deposits occurring predominantly in a periarticular location generally on the extensor aspect.[3,4] It locates mostly in the soft tissues of the hips, pelvis, shoulder, elbow joints, and less commonly in the wrist and ankle.[3,4] Knee involvement, especially localized on the flexor aspect is uncommon.[4] It generally occurs within the second or third decades.[1]

The etiology and pathogenesis is obscure. Smack *et al*. formulated a pathogenesis-based classification of the tumoral calcinosis into three types. (1) Primary normophosphatemic tumoral calcinosis; (normal levels of phosphate and calcium, sporadic cases). (2) Primary hyperphosphatemic tumoral calcinosis (elevated serum phosphorus and normal serum calcium, familial, and most common in the black race and males). (3) Secondary tumoral calcinosis: (chronic renal failure with secondary hyperparathyroidism, hypervitaminosis D, Milk-alkali syndrome, and bone destruction).[5]

The patient in the present case had no family history, biochemical abnormalities or underlying medical condition known, to promote calcification. The origin of primary normophosphatemic tumoral calcinosis (type 1 of the Smack classification) remains unknown, as the condition occurs sporadically and patients with the disorder have no abnormalities on laboratory examination.[5] Consequently, the present case was classified as type 1 of the Smack classification.

Our case is a sporadic case with relapses. Recurrence is common in familial and secondary cases, whereas, recurrence in sporadic cases is comparatively rare.[1,4,5]

Bone scintigraphy is a high sensitive method for determining the focus of tumoral calcinosis. The diagnostic radiological modality for tumoral calcinosis is generally direct radiography. In our case direct radiography had failed to detect the calcific deposit in the left iliac wing. In addition, it also helped us to determine the unknown foci, as it gave information about the whole body. It should be kept in mind as a differential diagnosis in patients with a mass seen in the soft tissue with dense calcification.
